# Supplementary material for: SIRT1 Activation Enhancing 8,3′-Neolignans from the Twigs of Corylopsis coreana Uyeki
Source: Plants (Basel). 2021 Aug 16;10(8):1684. doi: 10.3390/plants10081684 (PMC8398912; doi:10.3390/plants10081684)

<Supplementary data>

SIRT1 activation enhancing 8,3′-neolignans from the twigs of *Corylopsis coreana* Uyeki

Hyun Woo Kim [1,2], Jin Bum Jeon[1], Mi Zhang[1], Hyo-Moon Cho,[1] Byeol Ryu,[1] Ba-Wool Lee,[1] William H. Gerwick,[2,3] and Won Keun Oh [1,*]

[1] Korea Bioactive Natural Material Bank, Research Institute of Pharmaceutical Sciences, College of Pharmacy and Research Institute of Pharmaceutical Sciences, Seoul National University

[2] Center for Marine Biotechnology and Biomedicine, Scripps Institution of Oceanography, University of California, San Diego, La Jolla, California 92093, United States

[3] Skaggs School of Pharmacy and Pharmaceutical Sciences, UC San Diego, 9500 Gilman Drive, La Jolla, California 92093, United states

* Correspondence: wkoh1@snu.ac.kr

Contents

Figure S1. 1D NMR spectra of compound **1** in methanol-d_4

Figure S2. 2D NMR spectra of compound **1** in methanol-d_4

Figure S3. UV and ECD spectra of compound **1**

Figure S4. 1D NMR spectra of compound **2** in methanol-d_4

Figure S5. 2D NMR spectra of compound **2** in methanol-d_4

Figure S6. UV and ECD spectra of compound **2**

Figure S7. 1D NMR spectra of compound **3** in methanol-d_4

Figure S8. 2D NMR spectra of compound **3** in methanol-d_4

Figure S9. UV and ECD spectra of compound **3**

Figure S10. Cell viability of isolated compounds (**1-16**) on the HEK-293 cells

Figure S1. 1D NMR spectra of compound **1** in methanol-*d*$_4$

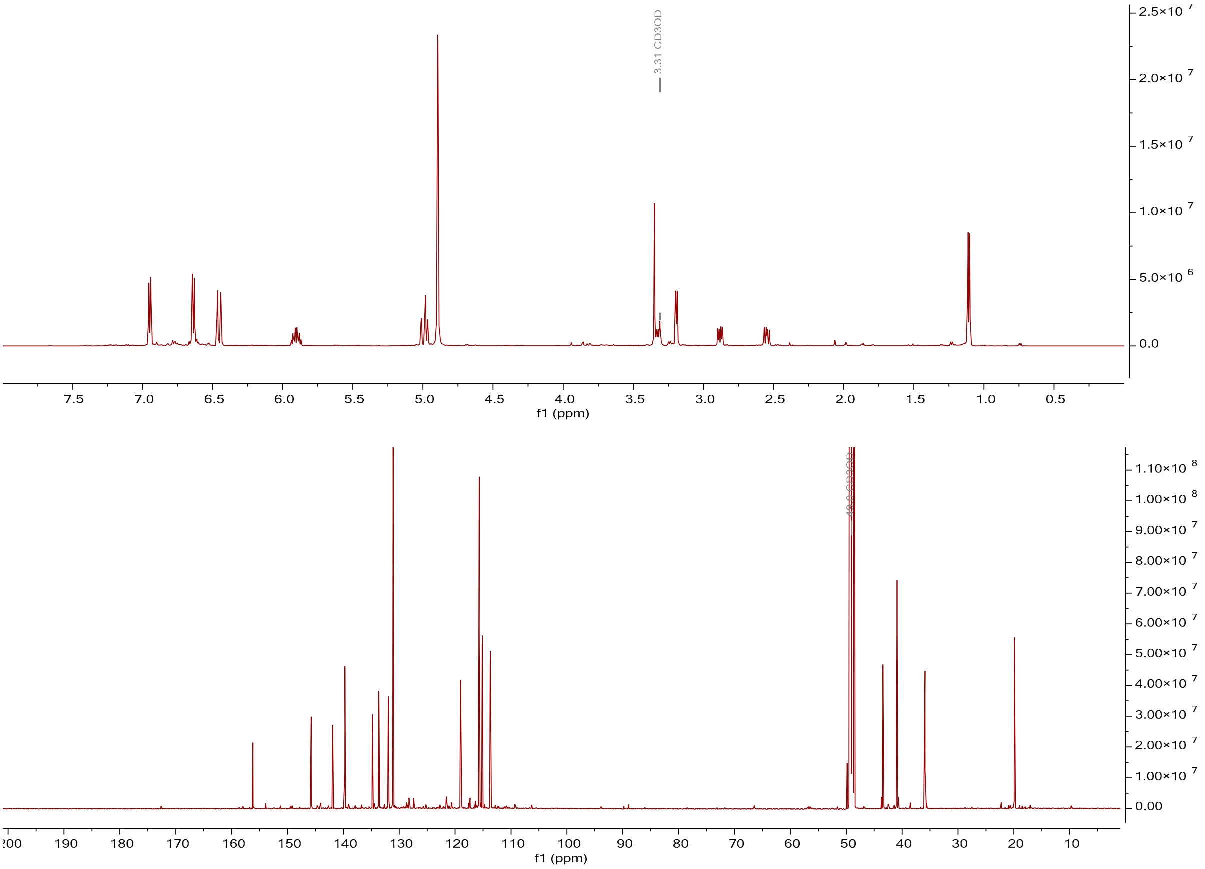

^{1}H and ^{13}C NMR spectra of compound 1

Figure S2. 2D NMR spectra of compound **1** in methanol-d_4

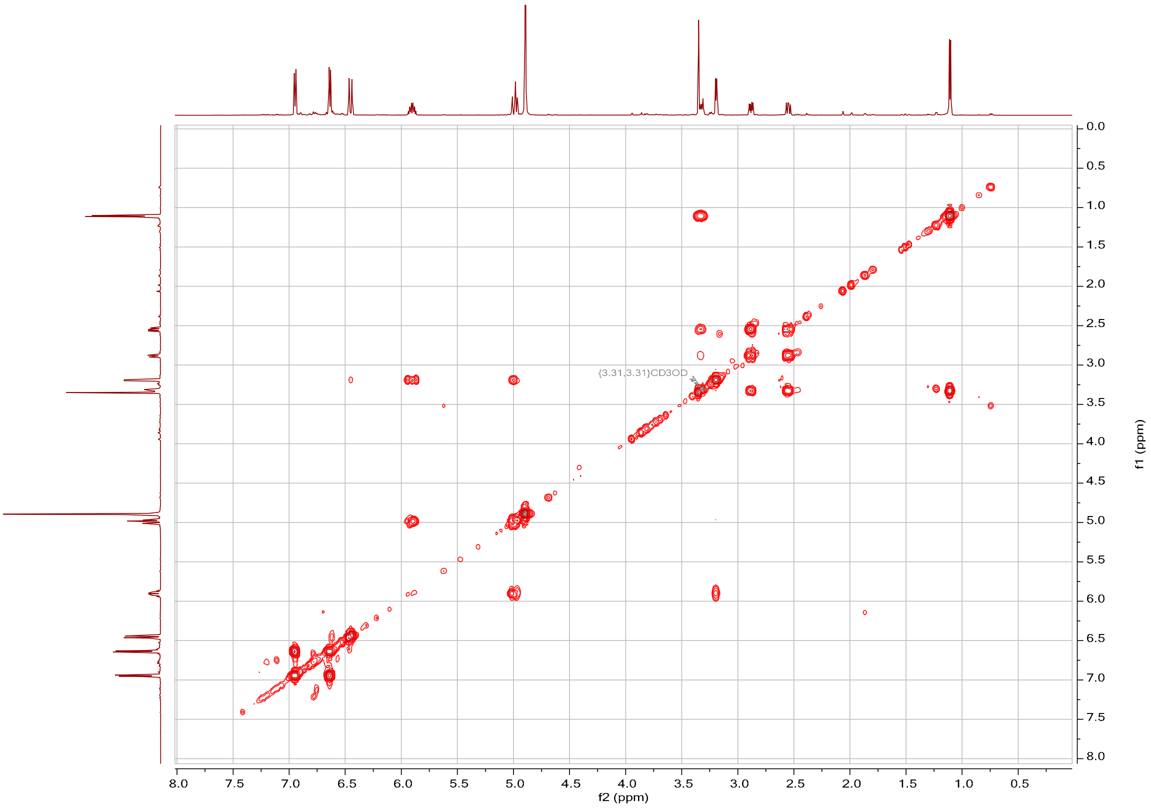

COSY spectrum of compound 1

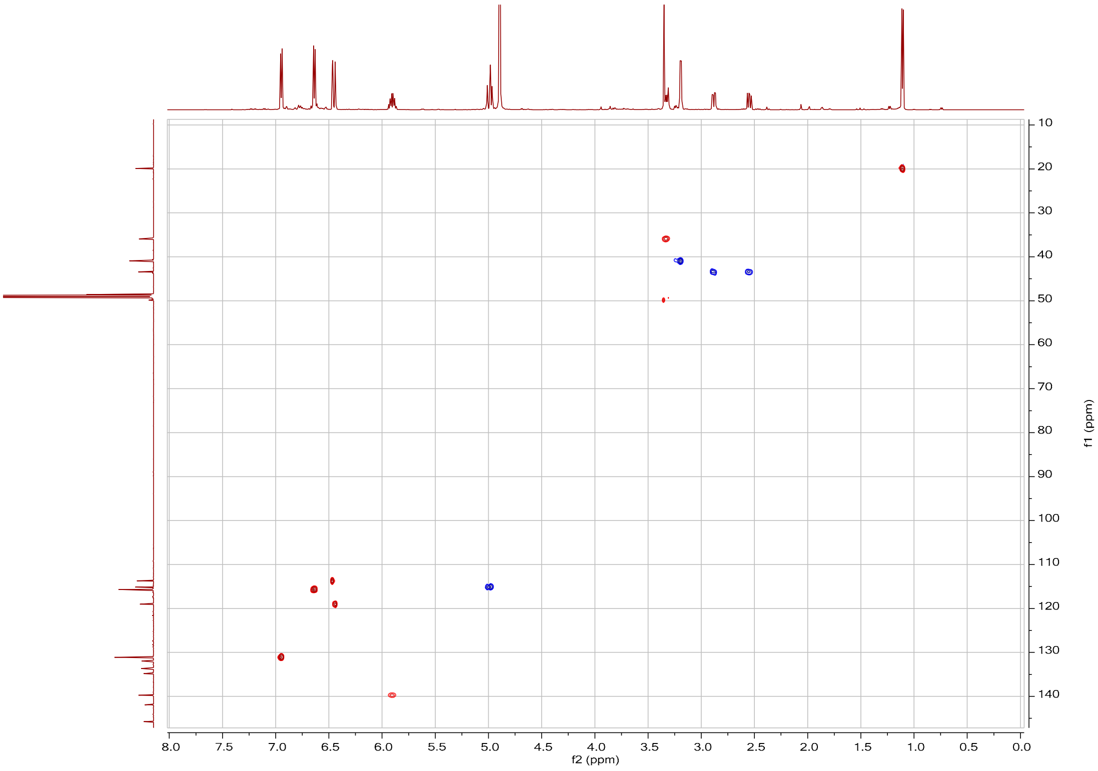

HSQC spectrum of compound 1

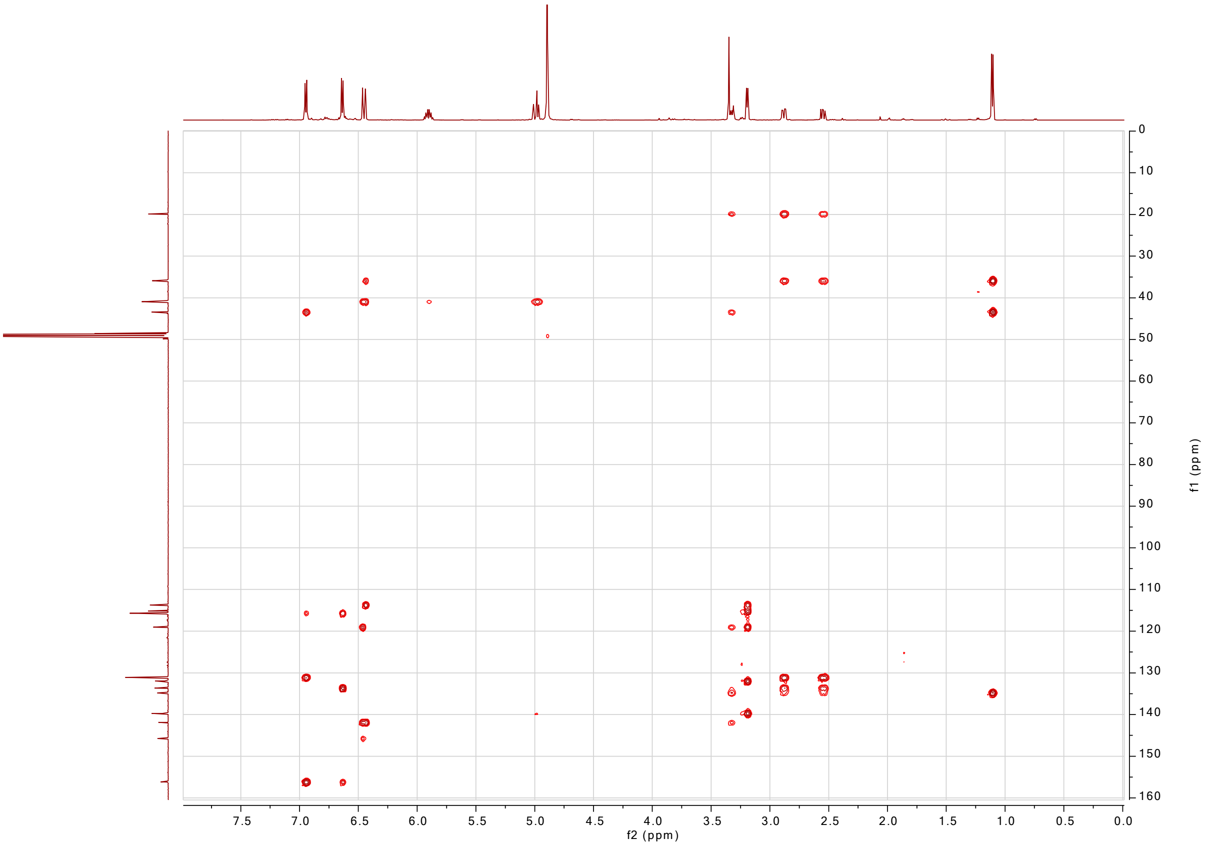

HMBC spectrum of compound 1

Figure S3. UV and ECD spectra of compound **1**

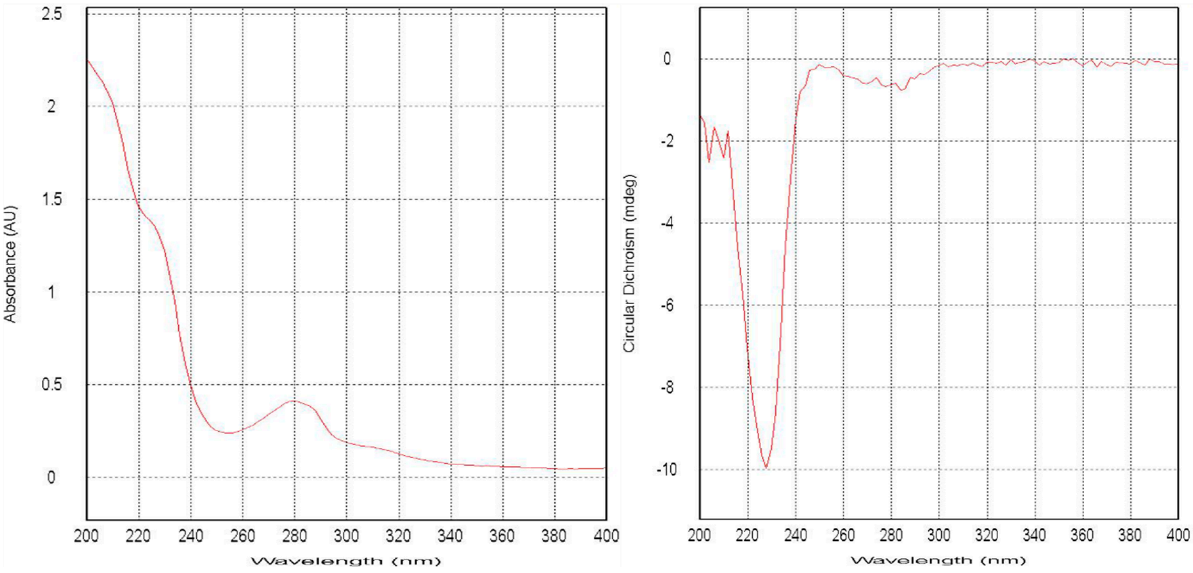

Figure S4. 1D NMR spectra of compound **2** in methanol- *d*$_4$

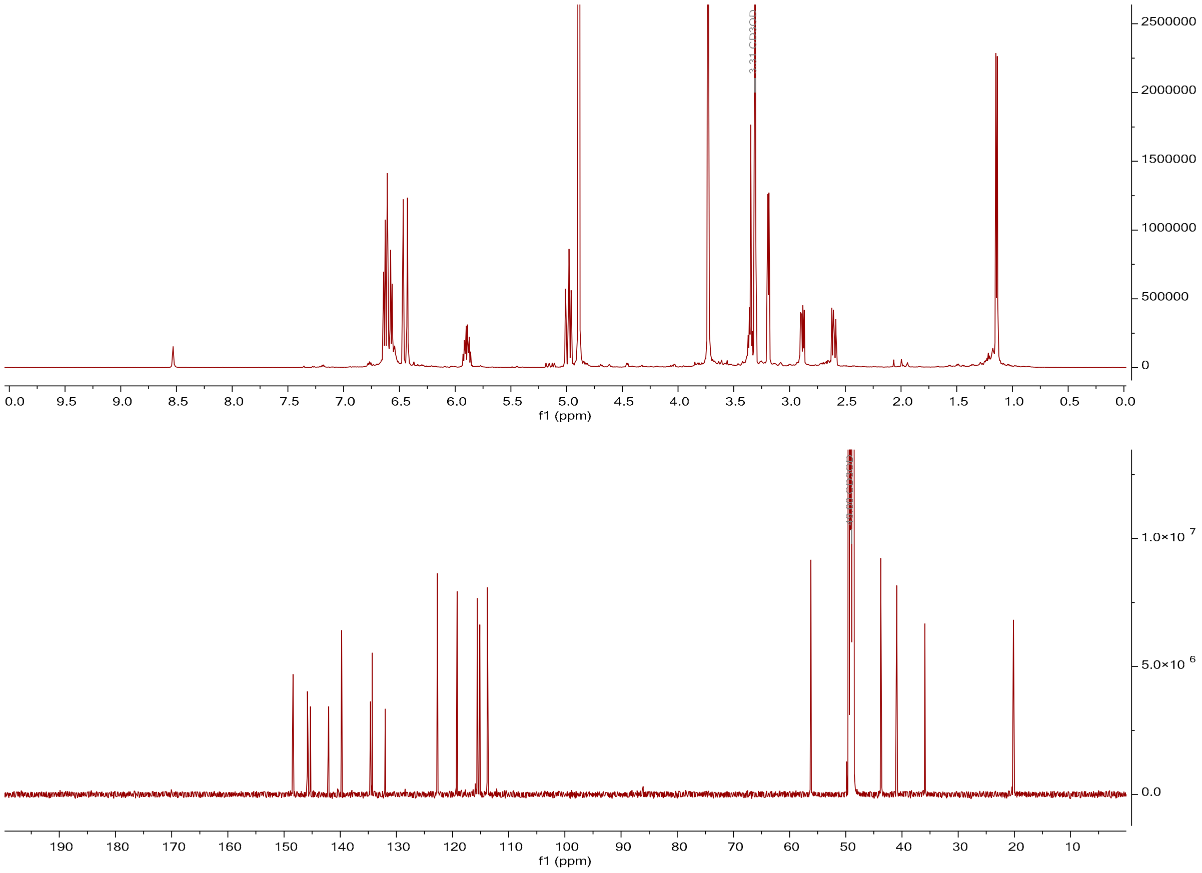

^{1}H and ^{13}C NMR spectra of compound 2

Figure S5. 2D NMR spectra of compound **2** in methanol-d_4

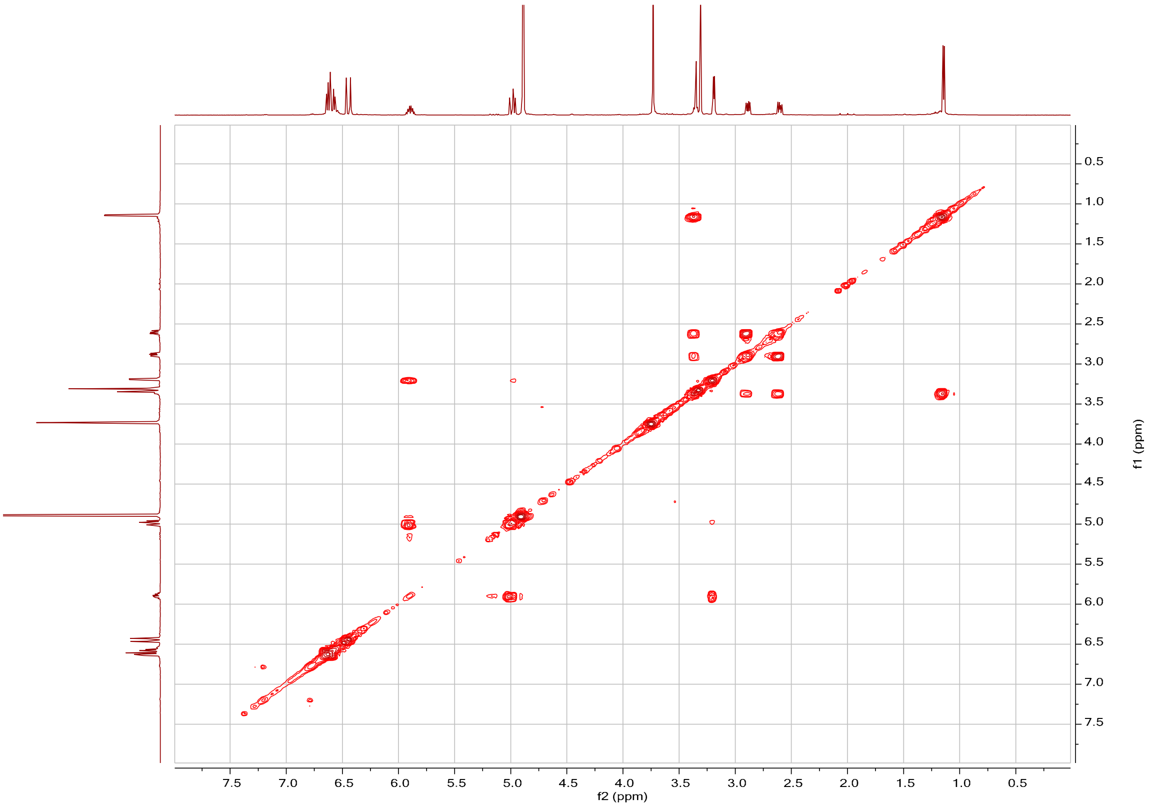

COSY spectrum of compound 2

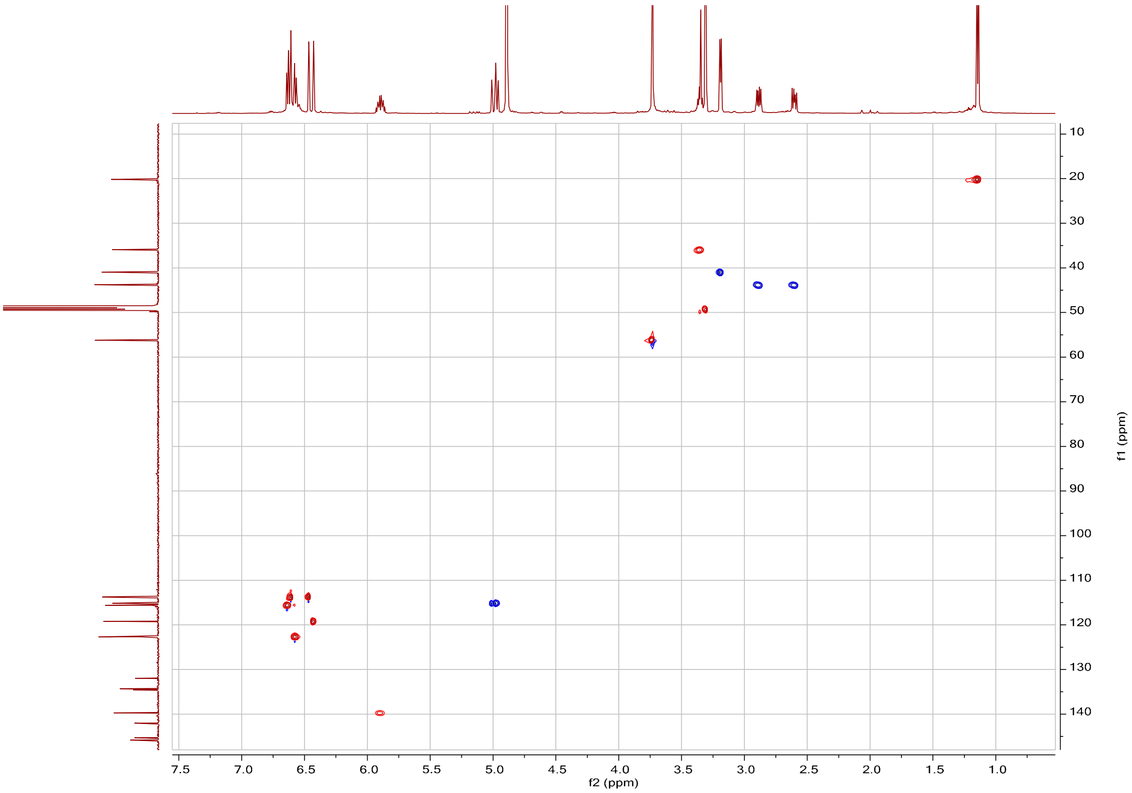

HSQC spectrum of compound 2

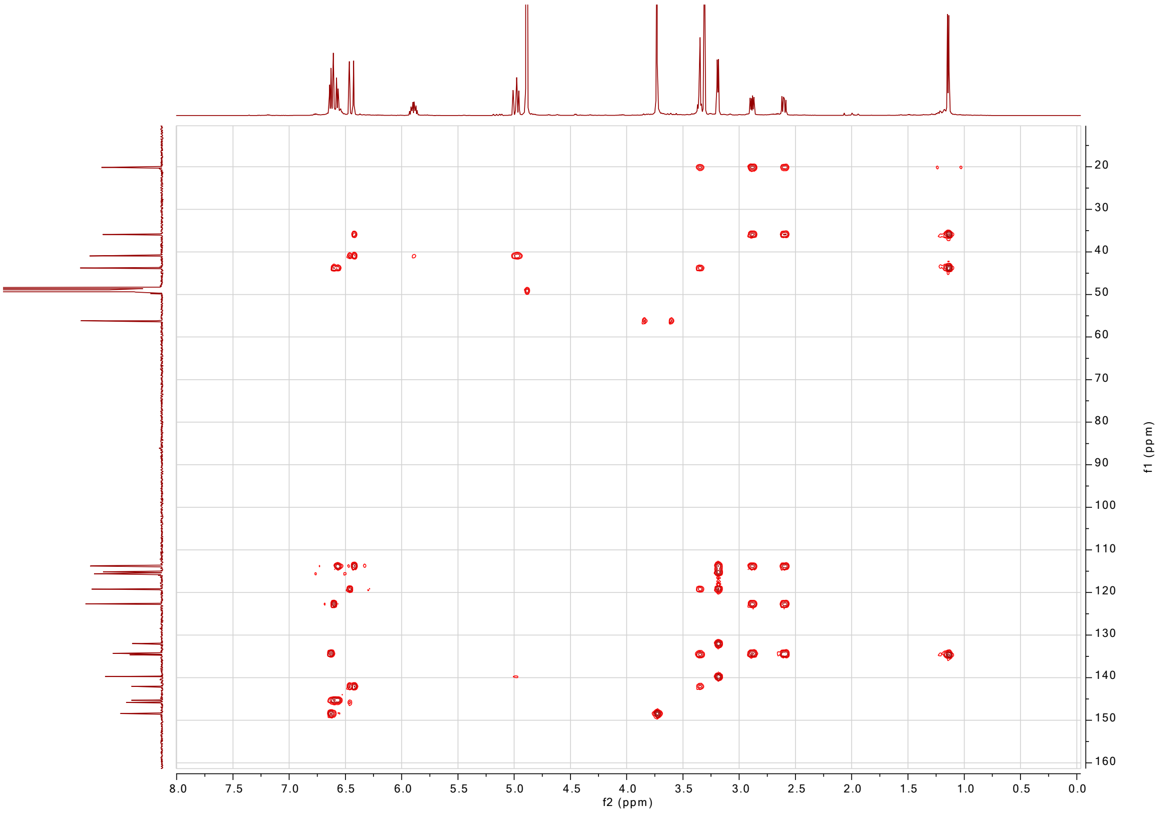

HMBC spectrum of compound 2

Figure S6. UV and ECD spectra of compound **2**

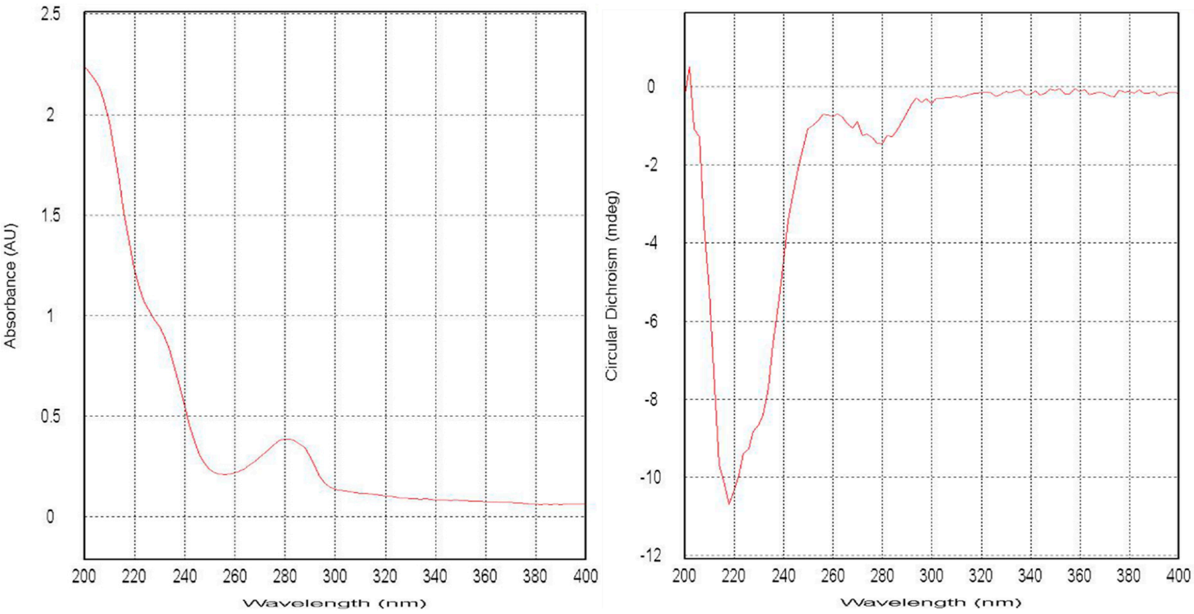

Figure S7. 1D NMR spectra of compound **3** in methanol-*d*4

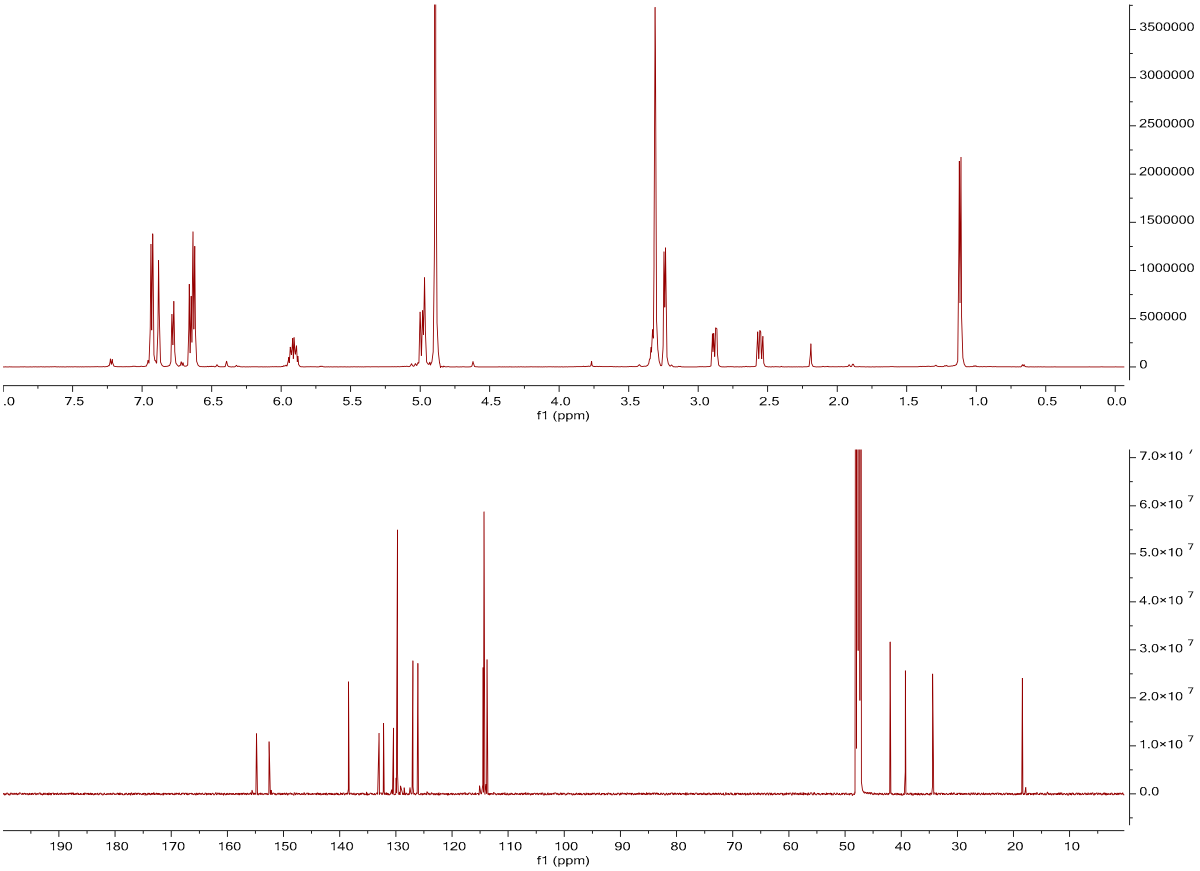

^{1}H and ^{13}C NMR spectra of compound 3

Figure S8. 2D NMR spectra of compound **3** in methanol-d_4

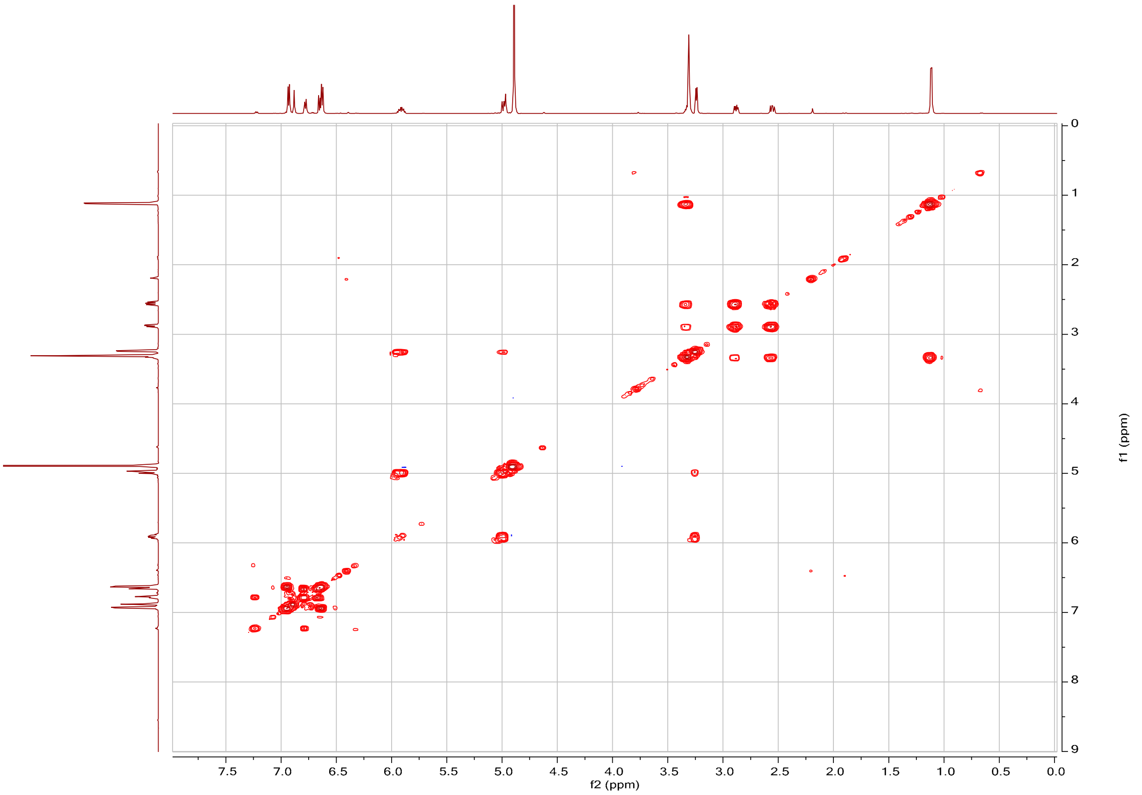

COSY spectrum of compound 3

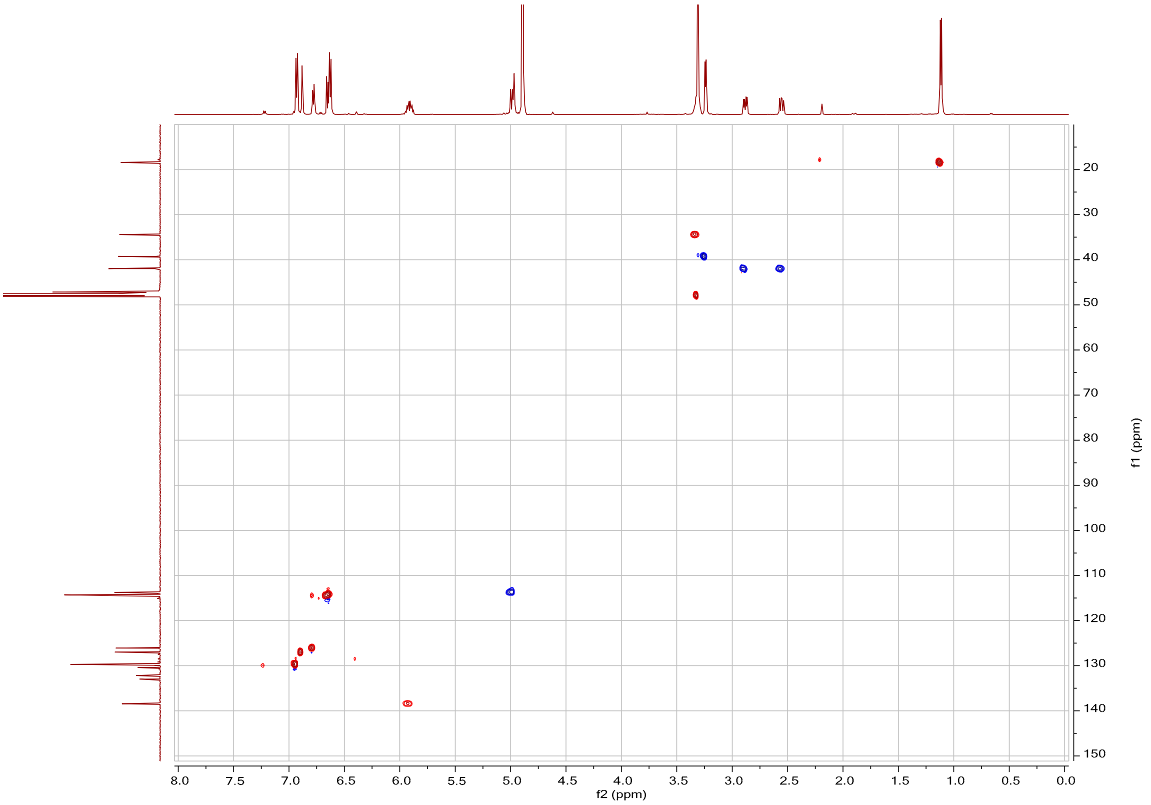

HSQC spectrum of compound 3

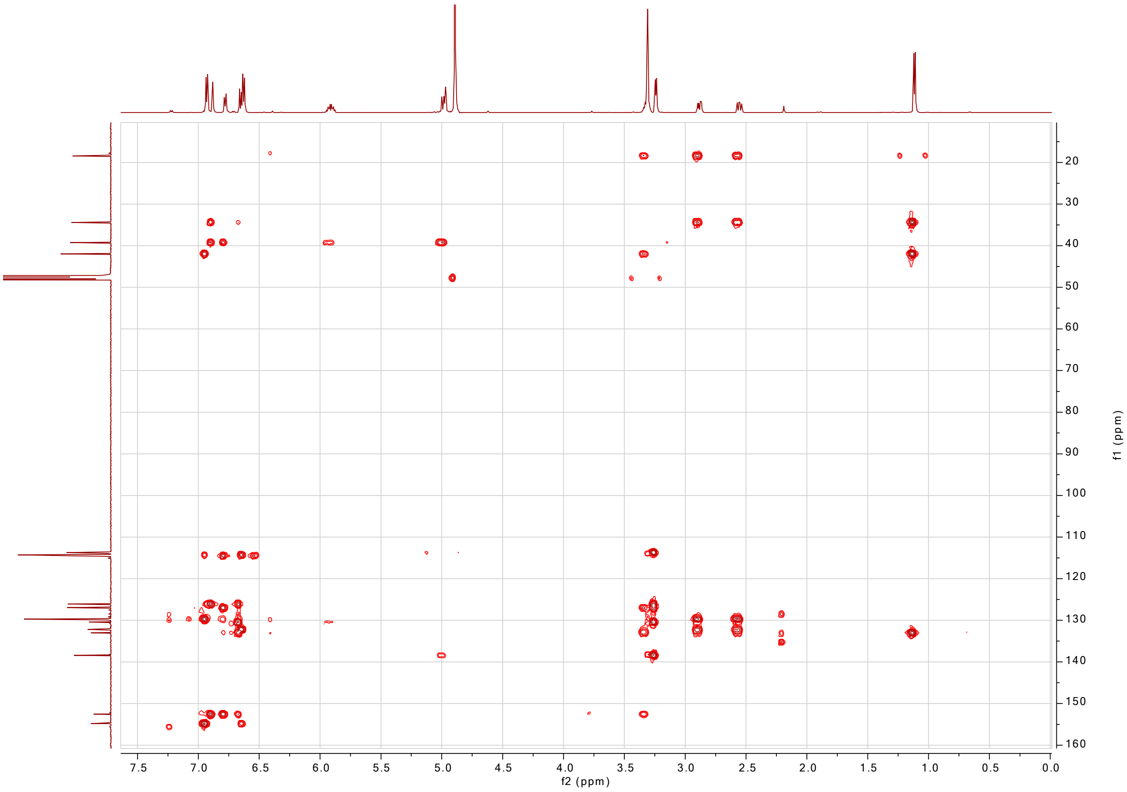

HMBC spectrum of compound 3

Figure S9. UV and ECD spectra of compound **3**

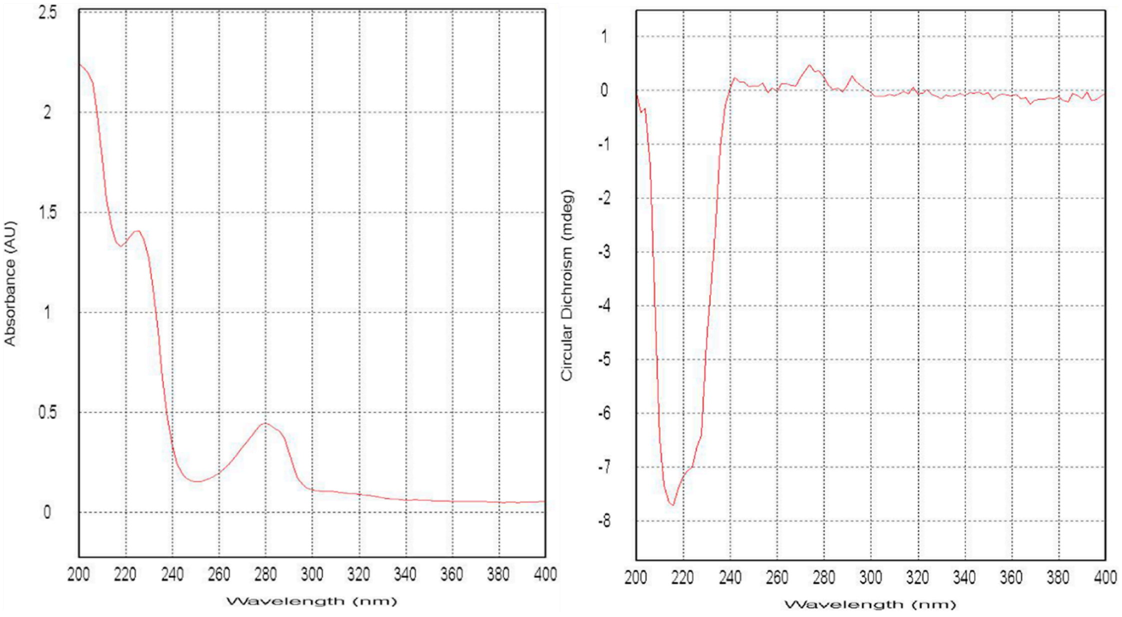

Figure S10. Cell viability of isolated compounds (**1-16**) on the HEK-293 cells

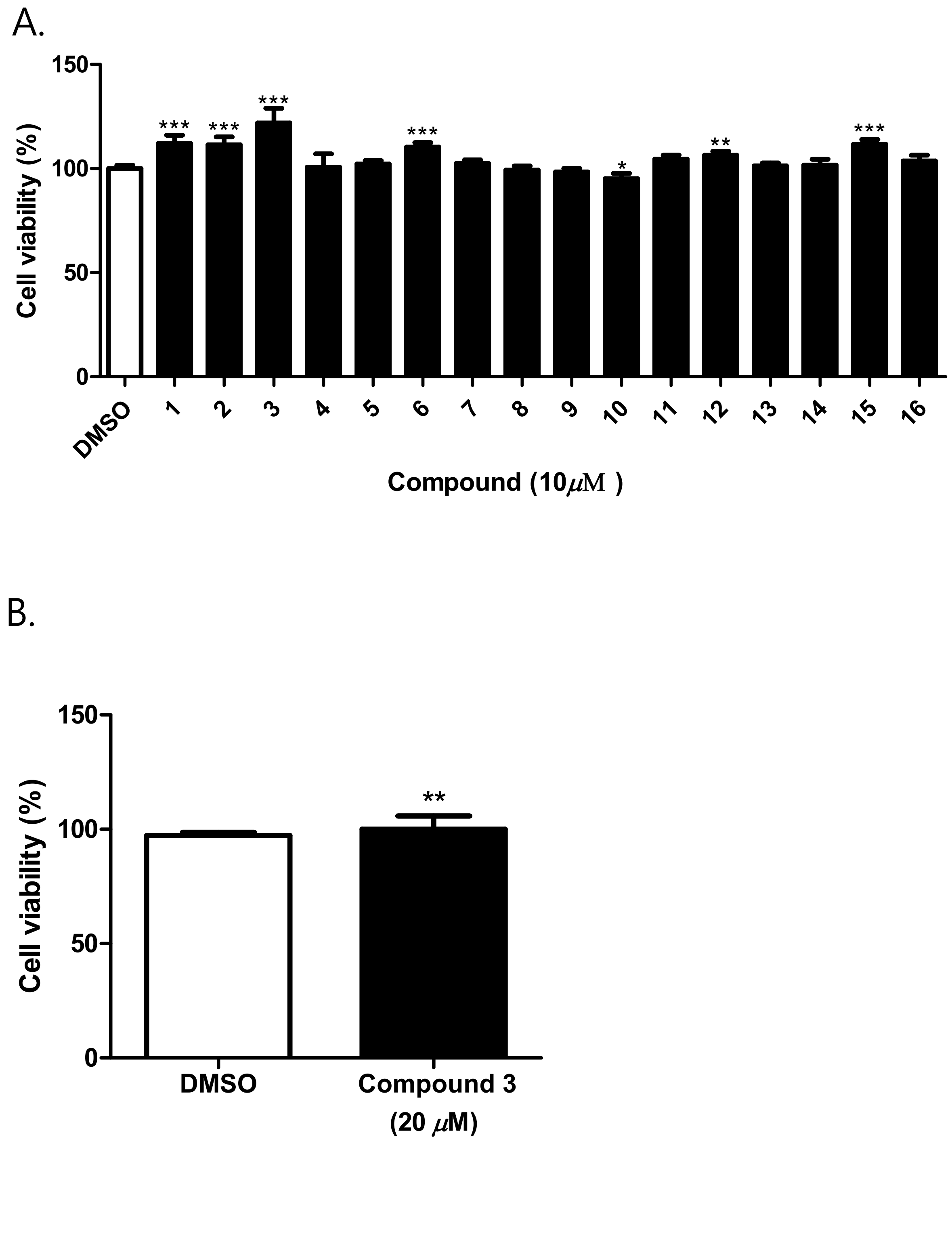

Supplement: Supplementary file 1 [file plants-10-01684-s001.zip › plants-1322929-SI.pdf]
